# Simultaneous Immunization with Multivalent Norovirus VLPs Induces Better Protective Immune Responses to Norovirus than Sequential Immunization

**DOI:** 10.3390/v11111018

**Published:** 2019-11-02

**Authors:** Maria Malm, Timo Vesikari, Vesna Blazevic

**Affiliations:** Vaccine Research Center, Faculty of Medicine and Health Technology, Tampere University, Biokatu 10, FI-33520 Tampere, Finland; maria.malm@tuni.fi (M.M.); timo.vesikari@tuni.fi (T.V.)

**Keywords:** norovirus, VLP, vaccine, genotype, pre-existing immunity, cross-reactivity, blocking antibodies, original antigenic sin (OAS)

## Abstract

Human noroviruses (NoVs) are a genetically diverse, constantly evolving group of viruses. Here, we studied the effect of NoV pre-existing immunity on the success of NoV vaccinations with genetically close and distant genotypes. A sequential immunization as an alternative approach to multivalent NoV virus-like particles (VLPs) vaccine was investigated. Mice were immunized with NoV GI.3, GII.4-1999, GII.17, and GII.4 Sydney as monovalent VLPs or as a single tetravalent mixture combined with rotavirus VP6-protein. Sequentially immunized mice were primed with a trivalent vaccine candidate (GI.3 + GII.4-1999 + VP6) and boosted, first with GII.17 and then with GII.4 Sydney VLPs. NoV serum antibodies were analyzed. Similar NoV genotype-specific immune responses were induced with the monovalent and multivalent mixture immunizations, and no immunological interference was observed. Multivalent immunization with simultaneous mix was found to be superior to sequential immunization, as sequential boost induced strong blocking antibody response against the distant genotype (GII.17), but not against GII.4 Sydney, closely related to GII.4-1999, contained in the priming vaccine. Genetically close antigens may interfere with the immune response generation and thereby immune responses may be differently formed depending on the degree of NoV VLP genotype identity.

## 1. Introduction

Noroviruses (NoVs) are the most common cause of epidemic acute gastroenteritis (AGE) in all age groups globally. NoV AGE leads to an estimated 212,000 deaths per year, mainly in young children in developing countries [1]. In developed countries, NoV AEG may cause deaths in the elderly and is associated with economic and societal costs [2]. The NoV has a single-stranded, positive-sense, RNA genome divided into three open reading frames (ORFs) that encode non-structural proteins (ORF1), a major structural capsid protein, VP1 (ORF2), and a minor capsid protein, VP2 (ORF3) [3]. NoV virus-like particles (VLPs) are spontaneously self-assembled by the main capsid protein, VP1, and can be produced in different expression systems for use as candidate vaccines [4,5,6]. Human NoVs belong mainly to genogroups (GI) I and II, which are further classified to genotypes GI.1–GI.9 and GII.1–GII.27 [7]. Approximately 90% of NoV outbreaks are caused by GII viruses, most belonging to the GII.4 genotype [8].

NoV particles bind in a genotype-specific manner to a versatile group of histo-blood group antigens (HBGAs) [9,10] that have been shown to be important for NoV entry and infection of the cells, functioning as attachment factors [11]. NoV blocking antibody assay, which measures the ability of antibodies to block the binding of VLPs to cell surface carbohydrates, HBGAs, is a surrogate for the standard neutralization assay [10,12,13,14]. Induction of blocking antibodies is one of the most important correlates of protection identified so far [15,16]. Blocking antibody responses to NoVs are largely genogroup-specific [12,17,18]. Variable levels of cross-blocking is observed between viruses inside the genogroup, depending on genetic and antigenic distance [16,19,20]. Thereby, even though >90% of children above five years of age have generated NoV-specific antibodies to several genotypes [14], repeated infections commonly occur [21,22,23]. The immune escape is most evident with predominant GII.4 genotype viruses that share >95% identity in their VP1 aa sequence [24]. Despite the close genetic relationship and pre-existing immunity to previously encountered strains, new GII.4 genotypes have emerged periodically every few years by epochal evolution of VP1 [20,25]. GII.4 genotype NoVs have caused seven pandemics since the mid-1990s, including Grimsby (1995/96 US), Farmington Hills (2002), Hunter (2004), Yerseke (2006a), Den Haag (2006b), New Orleans (NO) (2009), and Sydney (SYD) (2012) [25]. Since the 2012 GII.4 SYD pandemic, novel predominant GII.4 viruses have acquired different non-structural regions through recombination, but have retained the pandemic GII.4 SYD capsid [26]. Exceptionally, in the 2014–2015-winter season, concern over a global pandemic was raised when major non-GII.4 genotype, GII.17 Kawasaki outbreaks were reported on several continents, and GII.17 became the predominant genotype in several Asian countries [27]. Spread of the novel GII.17 strain was enhanced by lack of pre-existing GII.17-specific immune responses in the population and low cross-reactivity of GII.17 with other circulating NoVs [28,29].

As no cross-protective immunity exists between NoV genogroups, a multivalent vaccine or a bivalent VLP vaccine composition containing one GI (e.g., GI.1 or GI.3) and one GII genotype VLP (e.g., GII.4 or GII.12) is considered to be a minimum requirement [5,15,30]. The most advanced NoV VLP-based vaccine in clinical development is a bivalent vaccine containing GI.1 and GII.4 VLPs as a mixture [31]. Other vaccine candidates, combining two or more NoV VLP genotypes (a multivalent vaccine), have also been proposed [30,32], including a trivalent combination vaccine developed by our laboratory, containing a bivalent NoV VLP, GI.3, and GII.4-1999, and a rotavirus (RV) VP6 protein [5,32], targeted at two important causative agents of childhood acute gastroenteritis. Our NoV RV combination vaccine candidate is based on non-live subunit antigens and could improve the low efficacy observed with currently used live RV vaccines in developing countries [33]. RV VP6-specific IgA antibodies and CD4^+^ T cells have been associated with protection against RV infection [34,35].

The work described here investigates possible immunological interference among different VLPs combined as a mixture formulation with RV VP6. Also, sequential immunization as an alternative approach for NoV VLP vaccine immunization strategy has been explored.

## 2. Materials and Methods

### 2.1. Recombinant Proteins Production and Purification

**Figure 1:**
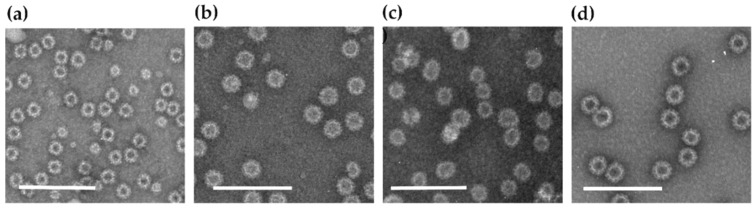
Electron microscopy images of the purified norovirus (NoV) genotypes: (**a**) GI.3, (**b**) GII.4, (**c**) GII.17, and (**d**) GII.4 SYD virus-like particles (VLPs). VLPs were examined by FEI Tecnai F12 electron microscope (Philips 487 Electron Optics, Holland) after negative staining with 3% uranyl acetate, pH 4.6. Bar, 200 nm.

Production and purification of NoV genotypes GI.3 (reference strain Genebank accession no: AF414403) (Figure 1a), GII.4-1999 (AF080551) (Figure 1b), GII.17 (BAR42289.1) (Figure 1c, and GII.4 SYD-2012 (AFV08795.1) (Figure 1d), as well as RV VP6 (GQ477131), used for immunizations, were produced in Sf9 insect cells by a recombinant baculovirus technology and purified in our laboratory as described in details previously [5,36]. VLPs used for analytical methods were produced either in a baculovirus expression system (GI.1-2001 and GII.4 NO-2010) [5,36] or in Nicotiana benthamiana plants (GI.4 and GII.4-2006 VLPs), as previously described [4]. The purity, integrity, and morphology of the VLPs were determined by SDS-polyacrylamide gel electrophoresis, immunoblotting, densitometric analysis, and electron microscopy (Figure 1a–d), as described elsewhere [5]. Protein concentration was determined by using a Pierce™ BCA Protein Assay Kit (Thermo Scientific, Rockford, IL, USA).

### 2.2. Mice Immunization

**Figure 2:**
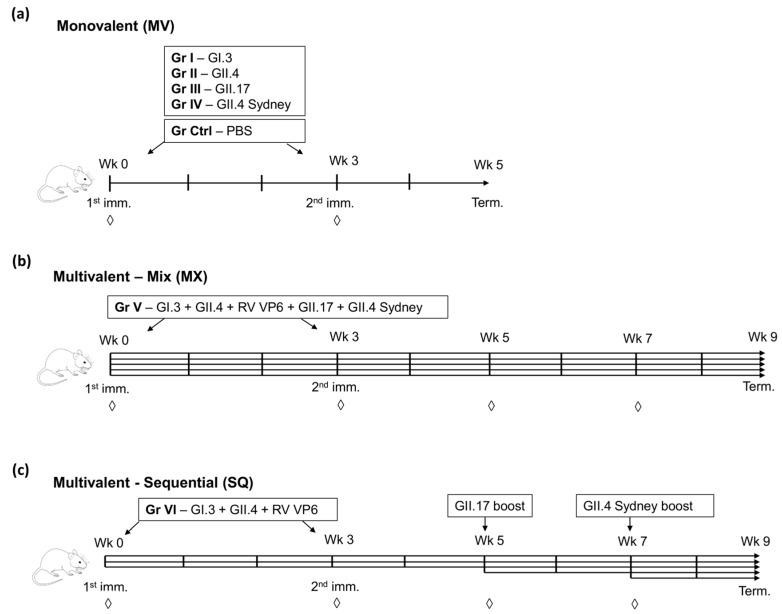
Immunization schemes of the study groups. Experimental groups of mice receiving monovalent (MV) (**a**) NoV VLPs (Gr I–IV) or the control group (Ctrl), receiving carrier only, were intramuscularly immunized twice at study weeks 0 and 3 and terminated at week 5. (**b**) Mice receiving a multivalent mix (MX, Gr V) of the four NoV VLPs and the RV VP6 protein was immunized using the same schedule but terminated at week 9. (**c**) Sequentially immunized mice (SQ, Gr VI) were primed twice at weeks 0 and 3 with the trivalent mix of NoV VLPs and RV VP6, boost immunized at weeks 5 and 7 with heterologous NoV VLPs, and terminated at week 9. Tail blood samples (◊) were collected at the indicated time points. Each horizontal arrow represents one injected antigen.

Female BALB/c (H-2d) mice were obtained from Envigo RMS BV and randomly divided into seven groups (Gr) of five mice (Figure 2). At seven weeks of age, the mice were immunized intramuscularly (IM) at the right caudal thigh muscle with a single, monovalent NoV VLP vaccine (Gr I–IV, Figure 2a), or as a multivalent formulation mixture of four NoV genotype VLPs and RV VP6 (Gr V, Figure 2b) two times, at week 0 and week 3 according to the established optimal immunization schedule used in our laboratory [5]. A 10 µg dose per each VLP was administrated, and control (Ctrl, Figure 2a) mice received phosphate-buffered saline (PBS) carrier only. The mice in the sequential immunization group (VI, Figure 2c) were primed with GII.4-1999 + GI.3 VLPs + RV VP6 (the trivalent NoV-RV combination vaccine [32]) at week 0 and week 3 and boosted with GII.17 VLPs at week 5, and with GII.4 SYD at week 7, using a 10 µg dose of each antigen per injection. Tail blood samples were collected at weeks 0 (pre-dose) and 3 from all mice and additionally at weeks 5 and 7 from the mice in the Gr V and Gr VI. Mice were sacrificed, and blood samples (serum) were collected at week 5 (Gr I-IV, Ctrl) or at week 9 (Gr V and VI). Immunizations were conducted under general anesthesia by inhalation of isoflurane (Attane vet, Vet Medic Animal Health Oy), and a formulation of medetomidine (Dorbene^®^ vet, Laboratorios Syva, Leon, Spain) and ketamine (Ketaminol^®^ vet, Intervet International B.V., Boxmeer, The Netherlands) was used for euthanasia. All procedures were carried out in accordance with the regulations and guidelines of the Finnish National Experiment Board (Permission number ESAVI/10800/04.10.07/2016) and mouse welfare was monitored throughout the experiment on a daily basis.

### 2.3. NoV-Specific ELISA

Homologous and cross-reactive serum IgG binding antibodies were detected by enzyme-linked immunosorbent assay (ELISA) as described elsewhere in detail [5,32]. In brief, individual mouse serum samples of the experimental groups and the control mice were analyzed two-fold diluted, starting at a dilution of 1:200. NoV- and RV VP6-specific IgG antibodies were detected with horse-radish peroxidase (HRP)-conjugated anti-mouse IgG (Sigma–Aldrich, Saint Louis, MO, USA) followed by o-Phenylenediamine dihydrochloride (OPD)-substrate (Sigma–Aldrich) and the optical density (OD) values were measured at 490 nm. The end-point titers of serum IgG were determined as the reciprocal of the highest dilution of serum, giving an OD above the set cut-off value (mean OD of negative control mice serum wells + 3 × SD) and at least 0.100 OD.

### 2.4. Blocking Assay

NoV VLP blocking assay, a surrogate neutralization assay, was used to determine the presence of serum IgG antibodies that block the binding of NoV VLPs to the HBGA carbohydrates according to the previously described method [37,38]. Pig gastric mucin (PGM, type III, Sigma–Aldrich, Cat. M1778) was coated on microwell plates (Corning Inc, Corning, NY, USA) and blocked with 5% milk in PBS. Starting serum dilution was 1:100 for homologous blocking and 1:20 for heterologous cross-blocking assay. Two-fold diluted serum samples were mixed and pre-incubated with NoV VLPs for 1 h at +37˚C prior to plating on PGM coated microwell plates. Bound VLPs were detected with anti-NoV polyclonal antisera (human [39] or rabbit [4]) followed by secondary IgG-HRP antibody (goat anti-human IgG, Novex, Invitrogen or goat anti-rabbit, Abcam, Cambridge, UK) and OPD-substrate (Sigma–Aldrich). Maximum binding was determined by VLP sample lacking mouse sera, and maximum binding OD 490 nm had to be ≥0.7 for each VLP to be acceptable [13]. The blocking index (%) was calculated as follows: 100% − [(OD490 of wells with VLP and serum/OD490 of maximum binding wells) × 100%]. Blocking titer 50 (BT50) was determined as the reciprocal of the highest serum dilution blocking at least 50% of the maximum binding.

### 2.5. Statistics

A non-parametric Mann–Whitney U test was employed to assess the statistical differences between observations of two independent groups. Multiple datasets were compared using a non-parametric Kruskal–Wallis test. Fisher’s exact test was used to assess the intergroup differences in the titers. Analyses were conducted by IBM SPSS Statistics (SPSS Inc., Chicago, IL, USA), Version 25.0 and with GraphPad Prism version 8 (GraphPad Software, La Jolla, CA, USA). The statistically significant difference was defined as *p* < 0.05.

## 3. Results

### 3.1. Simultaneous Immunization with Multivalent VLP Mixture Formulation

**Figure 3:**
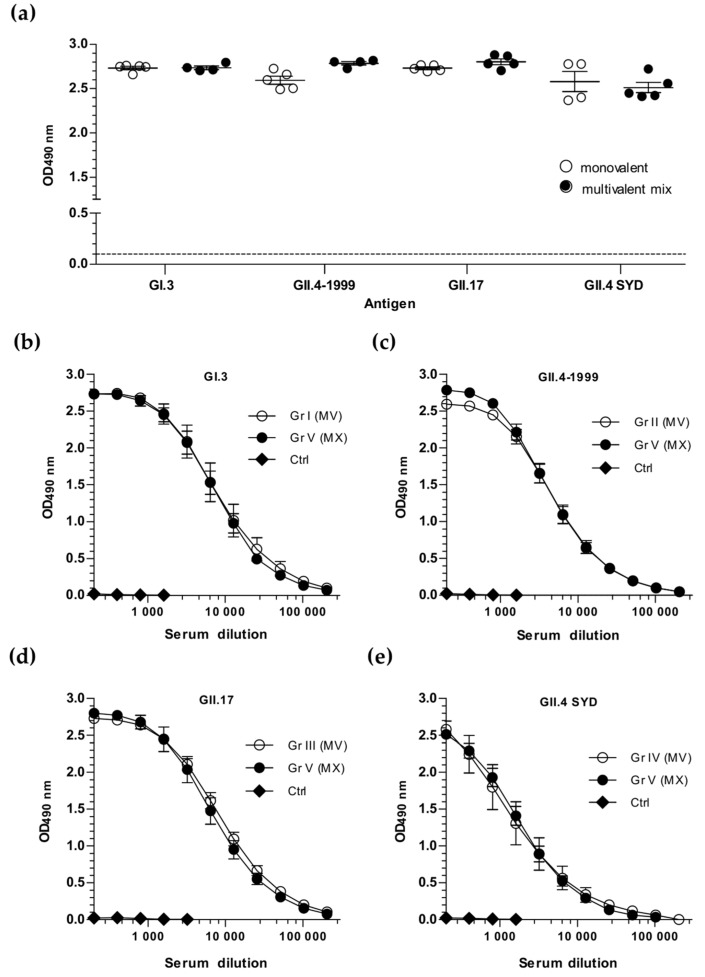
NoV genotype-specific IgG antibody responses induced by monovalent NoV VLPs or multivalent VLP mix immunization. Termination sera of mice immunized with monovalent (MV) VLPs, GI.3 (Gr I), GII.4-1999 (Gr II), GII.17 (Gr III), or GII.4 SYD (Gr IV), or with the multivalent NoV VLP mix (MX, Gr V), or the carrier only (Control group, Ctrl) were analyzed with enzyme-linked immunosorbent assay (ELISA). (**a**) The mean optical density (OD490) at the serum dilution 1:200 of individual mice is illustrated with group mean and the standard error of the mean (SEM). The horizontal dashed line indicates maximum background level (cut-off limit). Mean IgG end-point titration curves specific for NoV (**b**) GI.3, (**c**) GII.4, (**d**) GII.17, and (**e**) GII.4 SYD VLPs with the SEM are shown.

Similar NoV genotype-specific IgG binding antibodies were detected when comparing termination sera of mice immunized twice, either with 10 µg of each monovalent NoV VLP alone (Gr I-IV, Figure 2a) or as a component of a multivalent mixture (Gr V, Figure 2b) (Figure 3). No significant differences (*p* < 0.05) were observed when comparing genotype-specific IgG responses of monovalent or multivalent mix immunized mice at serum dilution 1:200 (Figure 3a). The results showed induction of equal levels of IgG antibodies against GI.3 (Figure 3b), GII.4-1999 (Figure 3c), GII.17 (Figure 3d), and GII.4 SYD (Figure 3e), irrespective of the presence or absence of other co-administrated antigens. NoV-specific IgG was not detected in any of the control animal sera (Gr VII) that received carrier (PBS) only (Figure 3b–e).

### 3.2. Sequential Immunization with Genetically Distant and Closely Related NoV VLPs

**Figure 4:**
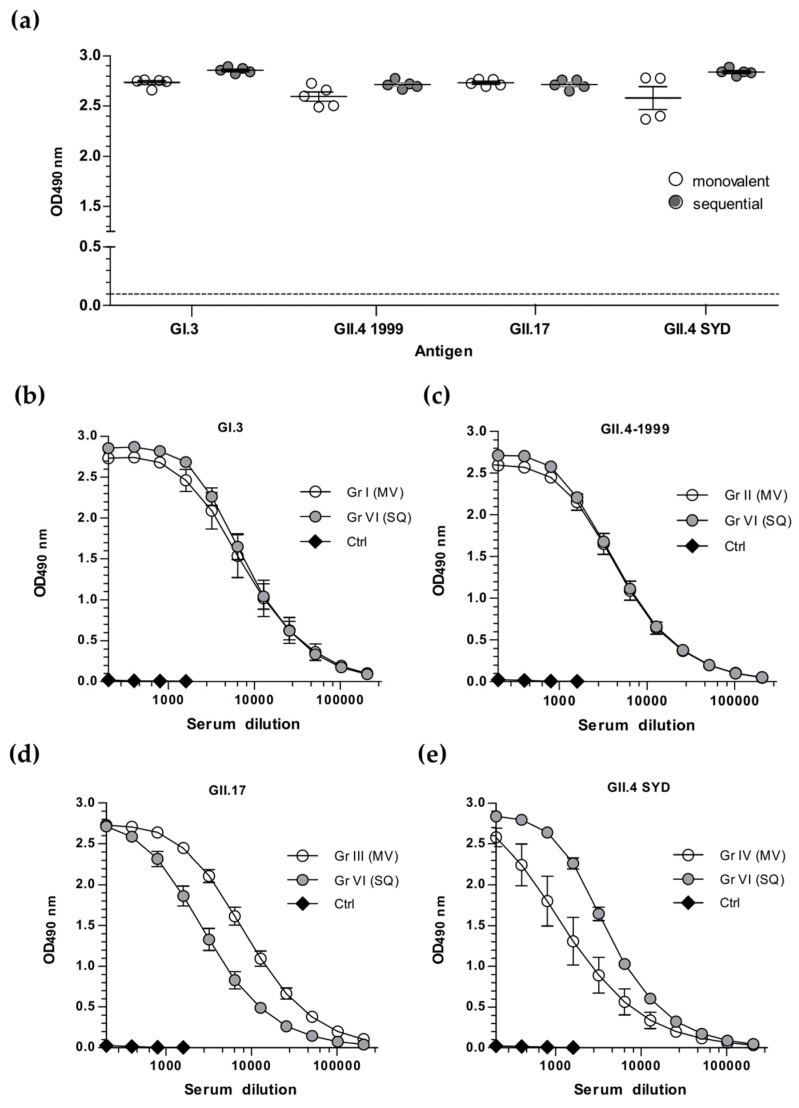
NoV genotype-specific IgG antibody responses induced by monovalent NoV VLPs or sequential VLP immunization. Termination sera of mice immunized with monovalent (MV) VLPs, GI.3 (Gr I), GII.4-1999 (Gr II), GII.17 (Gr III), or GII.4 SYD (Gr IV), or sequentially (SQ) immunized with heterologous VLP boosts (Gr VI) or the carrier only (Control group, Ctrl) were analyzed with enzyme-linked immunosorbent assay (ELISA). (**a**) The mean optical density (OD490) at the serum dilution 1:200 of individual mice is illustrated with the group mean and the standard error of the mean (SEM). The horizontal dashed line indicats maximum background level (cut-off limit). Mean IgG end-point titration curves specific for NoV (**b**) GI.3, (**c**) GII.4, (**d**) GII.17, and (**e**) GII.4 SYD VLPs with the SEM are shown.

As an alternative immunization strategy and to study the effect of pre-existing immunity, the sequential immunization schedule was employed (Gr VI, Figure 2). The mice primed twice (week 0 and week 3) with the trivalent combination vaccine formulation (GI.3 + GII.4 + RV VP6) [32] were further immunized with GII.17 VLPs at week 5, followed by a GII.4 SYD VLP boost at week 7, and termination sera IgG was analyzed for all four NoV genotype-specific IgG levels (Figure 4). When compared to genotype-specific immune responses of mice immunized twice with monovalent VLPs, no significant (*p* > 0.05) differences in IgG responses were observed (Figure 4a). Similarly, strong serum IgG titers to GI.3 (Figure 4b), GII.4-1999 (Figure 4c), GII.17 (Figure 4d), and GII.4 SYD (Figure 4e) were measured following one boost immunization, with no significant difference (*p* > 0.05) to corresponding monovalent immunization groups. The response to GII.17 (Figure 4d) was very strong considering that the mice received only one GII.17 VLP dose at week 5 (Gr VI). On the contrary, GII.4 SYD-specific IgG response in Gr VI mice sera (Figure 4e) consists of the genotype-specific antibodies as well as cross-reactive antibodies to GII.4 SYD induced by closely related GII.4-1999 VLPs.

### 3.3. Simultaneous and Sequential Immunizations Induce Different Level of Blocking Antibodies

**Figure 5:**
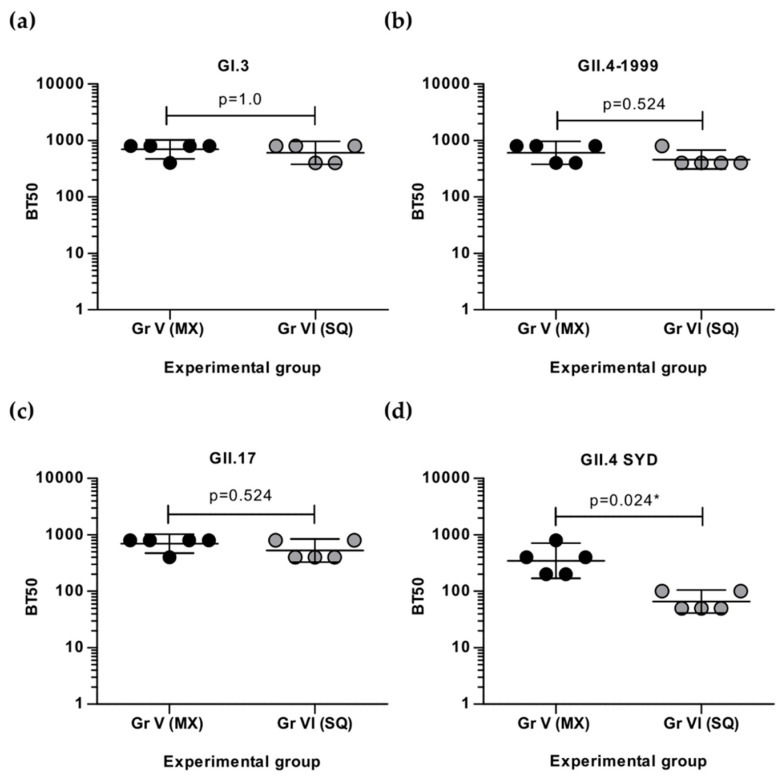
NoV genotype-specific blocking antibodies after immunization with multivalent NoV VLP as a mix or sequentially. Individual sera of mice immunized with multivalent mix (MX, Gr V) or sequentially (SQ, Gr VI) were 2-fold diluted starting at 1:100 dilution and assayed for the blocking of homologous NoV (**a**) GI.3, (**b**) GII.4-1999, (**c**) GII.17, or (**d**) GII.4 SYD VLP binding to histo-blood group antigens present in pig gastric mucin. The blocking index (%) was calculated as [100% − [OD (wells with serum)/OD(wells without serum, maximum VLP binding)] × 100%] and shown are individual mouse 50% blocking titer (BT50) and group geometric mean titer with 95% confidence interval. An arbitrary titer, BT50 of 5, was assigned to samples with <50% blocking index at the lowest serum dilution 1:100. Statistical differences were determined using Fisher’s exact test, and a *p* value of ≤0.05 was considered statistically significant (*).

To investigate if the neutralizing capability of IgG antibodies induced by simultaneous multivalent mixture immunizations (Gr V, Figure 2) is different from response to sequential immunization (Gr VI, Figure 2), genotype-specific blocking activity of the serum was analyzed. Equal levels of GI.3-specific blocking antibodies (Figure 5a) were generated by multivalent mixture (mean BT50 = 720) and sequential immunization (BT50 = 640). Similar levels of GII.4-1999-specific blocking antibodies were measured for both groups (mean BT50 = 640 Gr V; mean BT50 = 480 Gr VI) (Figure 5b). A single boost injection of GII.17 VLPs (Figure 5c) was sufficient to induce a comparable level of GII.17-specific blocking antibodies (mean BT50 = 560) to immunization with multivalent mix (mean BT50 = 720). In contrast, a single GII.4 SYD VLP boost injection (Figure 5d) generated significantly lower blocking antibodies (mean BT50 = 70) than immunization with multivalent mixture (mean BT50 = 400) (*p* = 0.024). Furthermore, the sera of mice immunized with the monovalent VLP vaccine (Gr I-IV, Figure 2) were tested for homologous blocking titers against all four genotypes (GI.3, GII.4-1999, GII.17, and GII.4 SYD) and blocking antibody levels similar to the levels induced by the multivalent VLP mix (Gr V) were observed (BT50 ≥ 400).

### 3.4. Cross-Protective Blocking Antibody Responses

**Figure 6:**
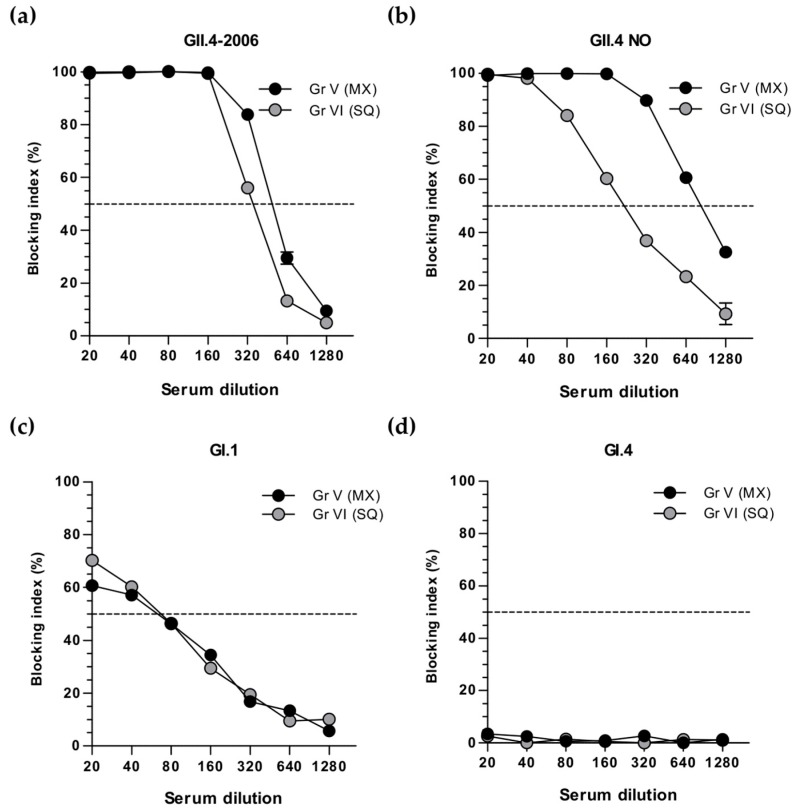
NoV cross-blocking antibodies after multivalent NoV VLP mix or sequential immunization. Group-wise pooled sera of mice immunized with multivalent mix (MX, Gr V) or sequentially (SQ, Gr VI) were 2-fold diluted starting at 1:20 dilution and assayed for the cross-blocking of heterologous NoV (**a**) GII.4-2006, (**b**) GII.4 NO, (**c**) GI.1, or (**d**) GI.4 VLP binding to histo-blood group antigens present in pig gastric mucin. The blocking index (%) was calculated as [100% − [OD (wells with serum)/OD(wells without serum, maximum VLP binding)] × 100%] and shown are group mean blocking indeces (%) with the standard errors of the mean of repeated assays. The horizontal dashed line represents a blocking titer of 50% (BT50).

The protective potential of serum antibodies induced by multivalent mixture (Gr V), or sequential immunization (Gr VI) against heterologous NoV VLPs belonging to GI (GI.1 and GI.4) or GII (GII.4-2006 and GII.4 NO) was tested by cross-blocking analysis (Figure 6). Blocking of GII.4-2006 VLP binding (Figure 6a) was equal in both groups (BT50 = 320). A considerable four-fold higher cross-blocking titer of GII.4 NO VLP binding was observed with multivalent mixture immunization sera (Gr V, BT50 = 640) than with sera of the sequential immunization group (Gr VI, BT50 = 160) (Figure 6b). Very low levels of GI.1 VLP cross-blocking antibodies were detected in both the Gr V and Gr VI immune sera (BT50 = 40) (Figure 6c), whereas no blocking antibodies to GI.4 VLP binding were observed (Figure 6d).

**Figure 7:**
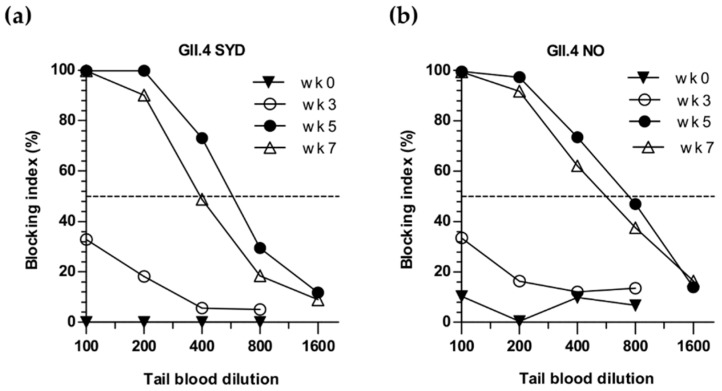
Kinetics of NoV type-specific and cross-reactive blocking antibodies after multivalent NoV VLP mix immunization. Group-wise pooled tail blood samples of mice were collected at the time of the first immunization (week 0), the second immunization (week 3), and two and four weeks after the second immunization (weeks 5 and 7) and 2-fold diluted starting at 1:100 dilution, for assaying the blocking antibodies of **(a**) homologous GII.4 SYD VLPs and (**b**) heterologous GII.4 NO VLPs binding to histo-blood group antigens present in pig gastric mucin. The blocking index (%) was calculated as [100% − [OD (wells with serum)/OD(wells without serum, maximum VLP binding)] × 100%] and shown are titration curves of each time-point blocking index (%). The horizontal dashed line represents a blocking titer of 50% (BT50).

Cross-blocking of heterologous GII.4-2006 (BT50 = 320) and GII.4 NO (BT50 = 640) VLPs binding by termination sera of mice simultaneously immunized with the VLP mix (Gr V, Figure 6a,b) was similar to genotype-specific blocking of GII.4-1999 (BT50 = 640) and GII.4 SYD (BT50 = 400) VLP binding (Figure 5b,d), respectively. To confirm this finding we measured the kinetics of blocking antibodies of Gr V to homologous GII.4 SYD (Figure 7a) and heterologous GII.4 NO (Figure 7b) in tail blood collected prior to immunization (week 0), after the first immunization (week 3) and two weeks after the second immunization (week 5). Low homologous (Figure 7a) and heterologous (Figure 7b) blocking activities were observed after the first immunization at week 3, followed by equally robust generation of blocking antibodies to GII.4 SYD (BT50 = 400) and GII.4 NO VLPs (BT50 = 400) following the second dose at week 5. Similar levels of blocking antibodies to GII.4 SYD (BT50 = 200) and GII.4 NO VLPs (BT50 = 400) were measured at week 7 (Figure 7a,b).

**Figure 8:**
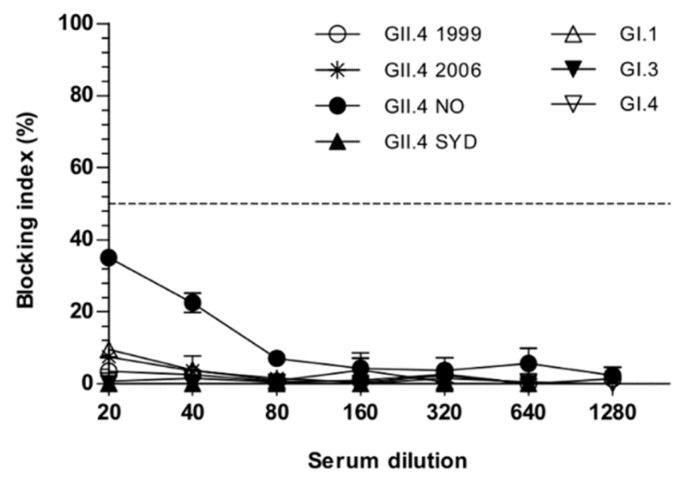
NoV cross-blocking antibodies after monovalent NoV GII.17 VLP immunization. Termination sera was pooled and serially 2-fold diluted starting at a 1:20 dilution and analyzed in a blocking assay against seven heterologous GI and GII VLPs. The blocking index (%) was calculated as [100% − [OD (wells with serum)/OD(wells without serum, maximum VLP binding)] × 100%] and shown are the blocking index (%) of the two analysis. The horizontal dashed line represents a blocking titer of 50% (BT50).

We also wanted to test the cross-blocking potential of GII.17 VLP immunized mice sera (Gr III, Figure 2), because it has been recently shown that GII.17-specific antibodies suppressed the replication of GII.4 SYD in intestinal epithelial cell cultures [40]. As shown above (Figure 3d, Figure 4d, and Figure 5c) monovalent GII.17 VLP immunization induced high genotype-specific IgG antibody levels and blocking antibodies; however, no cross-blocking antibodies to any of the seven VLP genotypes tested (GII.4-1999, GII.4-2006, GII.4 NO, GII.4 SYD, GI.1, GI.3, GI.4) were observed (Figure 8).

## 4. Discussion

The design of this study aimed to define the optimal immunization strategy with multivalent NoV VLPs and to predict the effects of pre-existing NoV immunity on the development of immune responses to novel NoV infections that may be caused by genetically very close or distant NoV genotypes. The basis for antigen selection and inclusion of RV VP6 into the multivalent mixture used in this study, and the sequential prime immunization, lies in the trivalent NoV-RV combination vaccine designed in our laboratory, consisting of NoV GI.3 and GII.4-1999 VLPs and RV VP6 protein [5,32,37,41]. Representative of GII, GII.4-1999 was selected as an ancestral genotype with a broad cross-blocking profile [20] and GI representative GI.3 VLP due to relatively high incidence in children [42]. By including RV VP6, the most abundant and highly immunogenic RV antigen, into the combination vaccine, this vaccine candidate is targeted to prevent two main causes of AGE in young children [5,32,37]. Even though the NoV VLP vaccine candidates tested in phase I/II clinical trials are either monovalent or bivalent VLP formulations [43,44], it is not known if it might be necessary to upgrade the vaccines according to emerging new variants, such as the sudden emergence of a novel non-GII.4 variant GII.17 in 2014 might suggest. Currently developed vaccines might not provide protection to GII.17 strains due to low cross-reactivity observed by ourselves and others [28,29].

We have comprehensively studied the immunogenicity of the trivalent NoV VLP and RV VP6 vaccine candidate in preclinical studies, demonstrating induction of strong NoV- and RV-specific immune responses [5,32,37,41]. No interference of the immune responses to any antigens of the combination vaccine have been observed when NoV- and RV-specific humoral and cellular responses have been assessed in mice immunized with bivalent [5] or trivalent [32] NoV-RV combination vaccine. Although VP6 induced immune response generation was not in the scope of the present study, both simultaneous and sequential immunizations induced similar VP6-specific antibody levels (data not shown). We have previously shown that NoV VLPs are highly immunogenic delivered either IM, intradermally (ID), intranasally (IN), or primed IM and boosted IN [45]. Here, we have extended our investigation of different NoV VLP vaccine delivery strategies [17,39] by combining four different NoV genotypes, two GII.4 VLPs, an ancient and a recent pandemic strain (GII.4-1999 and GII.4 SYD), one recently emerging non-GII.4 VLP (GII.17), and one GI genotype (GI.3). To determine the requirements for delivery of the multivalent vaccine antigens, we used NoV VLPs as a mixture delivered at the same time or at separated times, using sequential immunization. The results show that multiple NoV genotype VLPs can be delivered simultaneously as a multivalent VLP vaccine mixture (a cocktail) and immune responses to all vaccine antigens are induced. Congruent to our earlier findings [39], there was no inhibition observed by multivalent mixture immunization, as IgG binding and blocking antibodies were at an equal level to the responses induced by monovalent VLPs. This is in contrast to a previous report by Leroux–Roels et al. [44] showing that GI.1 VLP equivalent dose in a bivalent GI.1 + GII.4 VLP vaccine formulation interfered with GII.4-specific immune responses in humans. It could be speculated that the discrepancy here might be related to differences between NoV genotypes regarding the immunological interference they might possess in relation to other genotypes.

We also investigated if NoV-specific immune responses induced by vaccination may be differently formed depending on the degree of VLP genotype identity with NoV VLPs previously encountered. In the sequential immunization schedule, it could be assumed that the priming of mice with a combination of NoV GI.3 and GII.4 VLPs and RV VP6 mimics the baseline pre-existing immunity to these viruses, which exist in the pediatric population [22,46]. In addition, the boosting of mice with the GII.17 and GII.4 SYD VLPs at later time points reflects the children’s exposure to novel NoVs. The sequential immunization with NoV VLPs derived from genetically distant GII.17 genotype was successful in inducing high NoV GII.17-specific immune response. These results are in line with our earlier studies showing that sequential immunization with NoV VLPs derived from genetically diverse GII.4 and GII.12 genotypes has been successful in inducing high NoV-specific immune response to both genotypes [47]. In contrast, sequential boost immunization with GII.4 SYD VLPs, genetically closely related to GII.4-1999 (41% capsid protein identity, by the Protein Basic Local Alignment Search Tool) failed to induce strong blocking GII.4 SYD-specific responses when compared to mice immunized with multivalent VLPs mixed simultaneously. Noteworthy, even though similar level of GII.4 SYD-specific IgG titers were measured in all immunization groups, the GII.4 SYD blocking activity of sequentially immunized group sera was significantly lower, addressing the importance of measuring blocking NoV-specific IgG antibodies. The presence of blocking antibodies reveals the protective potential and specificity of these IgG antibodies, as previously reported in mice [17] and in humans [16], where the lack of cross-blocking antibodies was observed despite the presence of cross-reactive IgG antibodies. The lack of GII.4 SYD blocking antibodies in sequentially immunized mice was surprising, as it could be assumed that priming with closely related GII.4-1999 VLPs would increase GII.4 SYD-specific blocking antibodies. These results indicate that there might be an original antigenic sin (OAS) for closely related antigens (GII.4-1999 and GII.4 SYD, respectively), which could be an obstacle when considering frequently updated NoV VLPs vaccinations. Similarly, results in mice immunized sequentially with variant influenza viruses have suggested that OAS could be a potential strategy by which variant influenza viruses subvert the immune system [48]. Similarly, in humans, higher antibody titers to influenza strains encountered earlier in life following repeated exposures with new variants has been reported, indicating antigenic seniority [49]. Moreover, the antigenic distance hypothesis by Smith et al. in 1999 [50] suggesting that antigenic distance between circulating influenza strains and vaccine strains partly account for the variable vaccine efficacy, has also been supported by recent report [51]. If the same phenomenon would apply to NoVs, it should be taken into consideration when designing vaccines for NoVs. The lack of cross-blocking between GI.1 and GI.4 and vaccine genotypes used in the present study was congruent with our previous results showing low or absent cross-blocking between, e.g., GI.1 and GI.3 NoV VLPs [17].

It has been recently published [40] that GII.17 VLPs induce antibodies able to inhibit GII.4 SYD genotype replication in in vitro cell culture models. For the above reason and as we observed that GII.17 VLPs were highly immunogenic even when delivered only once in a sequential regime, we assumed that GII.17-specific sera might efficiently block the binding of heterologous NoV VLPs not included in the vaccine formulation. Unfortunately, we did not observe such cross-blocking antibodies in mice immunized with GII.17 VLPs. The discrepancy in the results may come from the differences in the assays used to measure the cross-blocking activity. Sato et al. used a novel in vitro cell culture infection model while we employed the widely used ELISA-based blocking assay as a surrogate neutralization assay [10,12,15]. Sato et al. have also shown that GII.4 SYD–specific antibodies did not block GII.17 virus replication. Congruently, we have published that the ancestor GII.4-1999 VLPs are superior to more recent GII.4 variants in inducing cross-reactive responses, including the cross-blocking response to GII.17 VLPs [17,20,28,52]. On the other hand, we observed that when mice are immunized with the quadrivalent NoV VLP mixture containing GI.3, GII.4-1999 (an ancestor GII.4 variant), GII.4 SYD (the most recent pandemic variant), and GII.17, cross-blocking of GII.4 variants 2006 and 2009 (NO) was comparable to homologous GII.4-1999 and GII.4 SYD VLP blocking. These results indicate that a combination of GII.4 VLPs from the ancestral and the most novel variant could suffice to protect against most GII.4 NoV infections. This is a relevant observation as GII.4 NoVs cause over 70% of all NoV AGE worldwide [53].

The protective immunity may be evaded by small changes in the immunodominant epitopes and, therefore, NoV vaccine antigens might need periodic updates, similar to seasonal influenza vaccines. Instead of using multivalent VLP mixtures, one option is to use monovalent or bivalent NoV VLPs to prime the immune system and to then boost with the diverse monovalent VLP. However, this study indicates that OAS might interfere with the immune response generation for genetically closely related NoV genotype VLPs used as a boost. Furthermore, in this study, by combining an ancestor GII.4 (1999) and a novel GII.4 (SYD), it was possible to induce great cross-reactive responses to other GII.4 variants, indicating that vaccine-induced protection could extend to genotypes not included in the vaccine.
